# From Passion to Abyss: The Mental Health of Athletes during COVID-19 Lockdown

**DOI:** 10.3390/ejihpe13030047

**Published:** 2023-03-14

**Authors:** Liliana Pitacho, Patrícia Jardim da Palma, Pedro Correia, João Pedro Cordeiro

**Affiliations:** 1 Instituto Politécnico de Setúbal, Escola Superior de Ciências Empresariais, Centro de Investigação em Ciências Empresariais (CICE-IPS), 2910-761 Setúbal, Portugal; 2 INTEC—Instituto de Tecnologia Comportamental, 1600-772 Lisboa, Portugal; 3 Instituto Superior de Ciências Sociais e Políticas, Universidade de Lisboa, 1300-663 Lisboa, Portugal; 4 CAPP—Centro de Administração e Políticas Públicas, Instituto Superior de Ciências Sociais e Políticas (ISCSP), Universidade de Lisboa, 1649-019 Lisboa, Portugal; 5 Institute for Legal Research (UCILeR), University of Coimbra, 3000-018 Coimbra, Portugal

**Keywords:** lockdown, sport, stress, sleep disorders, subjective happiness, mental health, pay cuts

## Abstract

The outbreak and pandemic of COVID-19 forced people into extreme isolation and social distancing, with significant limitations on various activity sectors, including sports. This study aimed to assess the psychological health status of athletes during sports lockdown. Additionally, we intend to verify the mediating role of sleep disorders in stress perception and subjective happiness. Our sample was composed of 1492 Portuguese athletes from eight different team sports. During sports lockdown, athletes were found to have high stress levels and low subjective happiness levels and experience sleep disorders. Finally, these results conclude that sports lockdowns harm athletes’ psychological health and well-being. Pay cuts to athletes are an extra stress factor that exacerbate these adverse effects on psychological health. Finally, sleep is a mediator variable between stress perception and subjective happiness levels. This study’s significant contributions, limitations, and future directions are discussed in the conclusion.

## 1. Introduction

An outbreak of the etiological agent SARS-CoV-2, leading to the new coronavirus (COVID-19), occurred in December 2019 in Wuhan, China. This outbreak quickly spread worldwide [1]. On 11 March 2020, the World Health Organization (WHO) registered more than 118,000 confirmed cases in 114 countries out of 196 worldwide and 4291 deaths. These data alarmed the authorities, and the WHO declared the virus a pandemic [2]. Without a vaccine or known medical treatment for the disease, unprepared countries’ governments adopted mandatory lockdown measures to stop the virus’s spread and protect the most vulnerable [3].

During the uncertain times caused by the current pandemic, undesirable stress levels were reported, as well as a breakdown in individual perceptions of well-being [4]. These effects can be exacerbated by lockdowns, which restricts relations with others and social contact or support [5]. The crisis caused by SARS-CoV-2 had severe implications for several sectors of activity [6]. This new and unexpected scenario, and the resulting stay-at-home policies, impaired the general population’s mental health, increased stress and anxiety levels and elevated the risk of depression and sleep disorders [7]. Although the literature on the impact of lockdown and sports interruptions on psychological health is still scarce, there is research evidence indicating that athletes show a higher risk of mental problems than the general population [8].

While the WHO encouraged the world population to undertake daily physical exercise to maintain their well-being and mental health [9], athletes were restricted in their usual daily activity, comprising several hours of physical activity. Worldwide, we witnessed the cessation of numerous activities, including sports. The world witnessed the postponement of the Olympic Games and the European Football Championship for the first time in history (UEFA EURO 2020). In Portugal, different sports’ championships were suspended or canceled, with corresponding interruptions to promotions and relegations in sports leagues. Lockdown potentially increases athletes’ vulnerability to negative mental health symptoms, and the pandemic situation presented extra challenges for athletes [10]. For example, with these changes in routine and the effects of social isolation, athletes have faced career disruptions and uncertainty regarding the status of their sports contracts. Additionally, athletes have had to deal with limited, and mostly denied, access to an effective training environment, as well as uncertainties regarding qualification processes due to the cancellation or postponement of significant national and international competitions [11]. All these limitations and uncertainties caused by the pandemic form barriers to pursuing athletes’ goals, whether these goals are amateur or professional, individual or team-based [12].

Sport is the central activity in the lives of many athletes. For some, this interruption represented the first time they were deprived of the ability to participate and compete in sports, resulting in high stress levels and other adverse psychological health effects [8,13]. COVID-19 and lockdown are a “longitudinal, multifaceted, unpredicted and non-controlled change-event” and represent a significant career disruption that could potentially affect athletes’ lives and career trajectories [14]. Previous studies demonstrate that events of change with these characteristics can be accompanied by negative feelings, identity crises, and the highest levels of distress. Sports lockdowns due to COVID-19 can be seen as career disruptors and promote a loss of identity, motivation, and meaning [15]. In another study, in response to the postponement of the Olympic Games 2020, Olympic athletes demonstrated higher levels of stress, sleep disorders, fear of uncertainty, rumination, and feelings of loneliness [11]. These results are consistent with the study on Italian athletes who could not maintain their sports training and competition routine, who showed an increase in stress levels and a dysfunctional psychosocial state [16].

During the lockdown, since stress manifests itself most readily in sleep disorders [17], sleep quality was impaired, and the subjects experienced a higher incidence of negative moods. Other authors state that people experience a slowing down of time in a lockdown situation, with increases in stress, anxiety, boredom, and sadness [18]. The relationship between stress and subjective happiness has been studied in two ways. On focuses on stress’ adverse effects on well-being and subjective happiness; the other suggests that subjective happiness can act as a buffer against stress [19]. Despite this two-way relationship, the negative effects of stress on happiness are widely demonstrated in the literature [20,21]. However, stress is not only related to happiness. Stress strongly predicts poor sleep [22]. The biopsychological consequences of stress can explain this significant relation. Stress promotes hyperarousal, which translates into elevated cortisol levels, increased heart rate and blood pressure, sympathetic activation, and inflammatory cytokines [23]. These biochemical levels are related to poor sleep quality and insomnia [24]. Moreover, some studies have shown that sleep also influences the subjective happiness of individuals. For example, sleep duration is associated with subjective happiness scores. A multi-country study showed that an increase in average sleep duration is associated with an improvement in subjective happiness score. Additionally, people with bad sleep quality showed lower subjective happiness levels, and recovery sleep is associated with physical and mental well-being [25]. Conversely, sleep deprivation is associated with less productive behavior [26]. Another study suggests that subjective happiness is related to the ability to fall asleep easily and better sleep efficiency [27].

Additionally, during interruptions to sport training and competitions, some athletes suffer monetary cuts. We propose that these cuts can compromise individuals’ most basic needs [28] and, as such, potentiate increases in stress and sleep disorders, as well as decrease levels of subjective happiness. According to the cognitive theory, when the loss, in this case, of financial resources, is appraised to be stressful, this can create a negative state of being, harm mental and physical health, and potentiate sleep problems [29]. Financial (in)security has implications for meaning in life. Meaning in life is an essential aspect of adaptive psychology and is related to subjective happiness and satisfaction [30]. Financial insecurity, represented in this study as monetary cuts, can promote non-adaptive functioning. Additionally, in previous studies, some authors verified that financial insecurity is a relevant factor for mental health during the COVID-19 pandemic [31].

This study has three main purposes: (1) to characterize the psychological health of athletes during the COVID-19 lockdown by analyzing stress, subjective happiness, and sleep disorders; (2) to perform a comparative analysis of the psychological health of athletes by reference to remuneration status and cuts; (3) to study the mediating role of sleep disorders on the relationship between stress and subjective happiness. To achieve these objectives, we hypothesize that, during interruptions to sport training and competitions:

**Hypothesis 1** **(H1).** *The athletes show high levels of stress.*

**Hypothesis 2** **(H2).** *The athletes show high levels of sleep disorders.*

**Hypothesis 3** **(H3).** *The athletes show low levels of subjective happiness.*

**Hypothesis 4** **(H4).** *The athletes feel unhappier than before the sports interruption.*

**Hypothesis 5** **(H5).** *Monetary cuts are associated with higher stress and sleep disorders and lower subjective happiness levels.*

**Hypothesis 6** **(H6).** *Stress negatively influences subjective happiness.*

**Hypothesis 7** **(H7).** *Sleep disorders mediate the relationship between stress and subjective happiness.*

## 2. Materials and Methods

### 2.1. Sample

Data were collected from a sample of 1.492 athletes, of which 45.2% were females. Participants ranged in age from 13 to 48 years (20.32 ± 6.72 years; mean ± standard deviation).

These athletes came from eight different team sports, namely, roller hockey (N = 319), basketball (N = 294), volleyball (N = 288), rugby (N = 248), futsal (N = 160), handball (N = 66), football (N = 64), and water polo (N = 53). The sample included two skill levels, the training level (727 participants) and the elite or competition level (765 participants). Training-level participants had an average age of 15.45 years (±1.19 years) and elite-level participant had an average of 24.94 years (±6.55 years). In this latter group, 22.6% of the athletes were paid salaries for their sports participation, and 12.7% were not paid salaries but received allowances. Some athletes experienced a cut in their remuneration and allowances during the lockdown. Of the 270 athletes who received a monetary income from sports, only 15.2% suffered no cuts, 32.6% suffered partial cuts, and most athletes suffered a total cut in their sports income (52.2%).

### 2.2. Procedures and Measures

The questionnaire was distributed and collected online through social networks and sports federations. Data were collected between 1 April and 30 May. Sociodemographic and sports questions were evaluated, and three scales were used: Perceived Stress Scale, Subjective Happiness Scale, and Sleep Disorders Scale. Further, we asked athletes about the regularity of their contact with sports clubs’ representatives and the psychological support level they provided.

#### 2.2.1. Perceived Stress Scale

The perceived stress was assessed by the 10-item version of the Perceived Stress Scale (PSS) [32]. All items were scored on a five-point Likert scale (0 = Never; 1 = Rarely; 2 = Sometimes; 3 = Often; 4 = Very Often). The reliability index of this scale in the present study was α = 0.83.

#### 2.2.2. Subjective Happiness Scale

Subjective happiness was assessed by the Subjective Happiness Scale (SHS) [33]. This scale is composed of four items and scored on a seven-point Likert scale; concerning reliability in this study, the SHS presented an α = 0.71.

Nevertheless, we considered it essential to measure the perception of changes in subjective happiness. A new independent item that evaluates the subjective happiness change (Happiness Change Item (HCI)) was added to the questionnaire. In this item, each athlete was asked, “How have you felt since the championship interruption”, and the answers were scored on a seven-point Likert Scale (1 = Much Less Happy; 7 = Much Happier). This is an independent item that was never performed with SHS; that is, this item does not integrate average subjective happiness.

#### 2.2.3. Sleep Disorders Scale

Sleep problems were assessed by the Sleep Disorders Scale (SDS), a subscale of the Copenhagen Psychosocial Questionnaire II (COPSOQII) [34]. This is composed of four items and was scored on a four-point Likert Scale (1 = Never; 2 = Rarely; 3 = Sometimes; 4 = Often; 5 = Always). In this study, the scale reported a reliability index of α = 0.81.

### 2.3. Statistical Analysis Procedures

Regarding data preparation, no missing data were found, as all survey questions were mandatory. The software SPSS statistics (V. 27 IBM SPSS) was used in all analyses. Firstly, descriptive statistics were calculated as measures of central tendency and dispersion (mean, standard deviation, and maximum and minimum value) for each variable in the study. Association measures were also calculated. Namely, the Pearson correlation coefficient (r) was used, with a 99% confidence interval. The comparative method was used to compare the levels of stress, subjective happiness, and sleep disorders between paid and unpaid athletes, as well as between the different types of pay cut. A non-parametric comparative test (Kruskal–Wallis) was performed because the assumptions of normality of distribution and homogeneity of variances were not met. Then, the averages of the orders were compared using the error type I (0.05).

Lastly, to analyze the mediation hypothesis, a linear regression was performed using the Enter method, and the assumptions of independence and multicollinearity were checked through Durbin–Watson Statistic and VIF. Then, the macro-PROCESS for SPSS was used, specifically Model 4, which postulates a mediation model with a mediating variable. This method was used as an analytic strategy to evaluate the indirect effect of stress perception level (X) on subjective happiness (Y) through the mediating process of sleep disorders (M). We calculated the indirect effect using 10,000 bootstrap samples for the bootstrap confidence intervals (CI) corrected for bias. An indirect effect is considered statistically significant if the established CI (CI at 95%) does not include a 0 value. If the 0 value is included in the CI, the null hypothesis establishes that the indirect effect equals 0; that is, there is no association between the involved variables [35].

## 3. Results

### 3.1. Descriptive and Correlational Analysis 

This first analysis revealed that, during sports lockdowns, the athletes’ mental health degraded and allowed for us to identify some risk factors (Table 1). The high stress values can revealed an adverse reaction to involuntary interruption. No values were obtained for comparison before the competition interruption, but we used the standard results found for the Portuguese population [36]. These authors studied the psychometric properties of PSS for the Portuguese population and established that scores above 20 points in the PSS represent a pathological result for men. Scores above 22 points are pathological in the case of women. Through a test of means comparison, it was verified that the mean of the sample in the feminine gender was significantly above the cut-off value (22 points) for pathology (t(671) = 6.584; *p* < 0.001). Moreover, the same is true for the mean of the sample in the male gender, which is significantly above (t(817) = 5.377; *p* < 0.001), the cut-off point (20 points). The sample mean in this study is higher than 22 points on the PSS, which shows exceptionally high values for this variable. Through frequency analysis and the standard values [36], we found that 61.1% of females and 41% of males showed pathological stress levels in the training-level group. In the competition-level group, 61.1% of women and 68.3% of men showed pathological stress levels. These results corroborate H1 (the athletes show high levels of stress).

To analyze sleep disorders, we used the national reference values to compare the results obtained in this study. The Portuguese population’s average reference value is 2.46 ± 1.05 in SDS. In addition to these reference values, this scale should score the level of risk to each individual’s health and, as such, follow the international indications for the application of the scale: values from 1 to 2.33 (green) correspond to a low level of health risk; values between 2.33 and 3.66 (yellow) correspond to moderate risk to health; values between 3.66 and 5 points (red) correspond to high risk to health [34]. The results of this study (3.39 ± 0.76) showed that the mean presented by the athletes in this study is higher than the standard for the Portuguese population and represents a moderate health risk. Additionally, we verified that only 12.2% of athletes in our sample presented low health risk indices, while 44.5% showed mean values representing a moderate health risk. Furthermore, a concern is that 43.3% of the athletes in this study presented a result above 3.66 points on the scale, representing a high risk for individual health. These results corroborate H2 (the athletes show high levels of sleep disorders).

Regarding subjective happiness, we verified that the sample average was 4.77 ± 1.04. As with the stress analysis, we did not have a previous comparative value. However, we compared this result with previous results, obtained in the original study of the SHS and other Portuguese samples. In the Portuguese validation of SHS, the authors obtained an average of 5.12 ± 1.02 [37]. In another study on Portuguese athletes, the author achieved an average of 5.41 ± 0.09 [38]. In the validation of the scale adapted for adolescents, also in Portugal, an average of 5.65 ± 1.05 was obtained [39]. When we compare our results with these previous studies, our study’s average subjective happiness is shown to be significantly lower. Comparing the results obtained in the training class (M = 4.94; SD: 1.06) with the average scale adaptation for young Portuguese people (M = 5.65: SD: 1.05), young athletes are shown to be significantly less happy than usual (t(726) = −17.989; *p* < 0.001). The same results were obtained when we compared the means between competition class in this study (M = 4.61; SD = 1.01) with the general Portuguese population before lockdown (M = 5.12; SD = 1.02) (t(765) = −13.989; *p* < 0.001), as well as with athletes (M = 5.41; SD = 0.09) (t(765) = −21.976; *p* < 0.001) before lockdown. We can, therefore, state that H3 (the athletes show low levels of subjective happiness) was corroborated, and that the athletes show lower levels of subjective happiness than before.

Furthermore, the results show that the athletes perceived a marked breakdown in their subjective happiness that was above and beyond the low average subjective happiness. They considered themselves significantly less happy than before this competition and training interruption. For this item, which varies between 1 (Much Less Happy) and 7 (Much Happier), the mean was 2.65 ± 1.17. In addition to this low average, we noted the response frequencies. We found that 75.7% of the sample athletes were less happy than before (between 1 and 3 on the measuring scale). We consider these percentages to be too high, and they represent a sharp drop in subjective happiness during this interruption. These results prove that H4 (the athletes feel unhappier than before the sports interruption) was corroborated and that team athletes feel unhappier than before the sports interruption.

Additionally, we analyzed the correlations between these variables. As expected, a significant positive correlation was verified between perceived stress and sleep disorders (r = 0.416). This means that more stressed athletes have more sleep disorders. Subjective happiness has a significant negative correlation with perceived stress (r = −0.461) and sleep disorders (r = −0.290). Athletes with higher stress levels and higher sleep disorders present low levels of subjective happiness. A significant negative correlation was found between perceived stress (r = −0.221) and sleep disorders (r = −0.083), with inherent changes in subjective happiness. The athletes who perceived a greater breakdown in their happiness also perceived higher stress levels and reported more sleep disorders.

Regarding H5 (monetary cuts are associated with higher levels of stress and sleep disorders and lower levels of subjective happiness), we compared athletes that either received or did not receive remuneration or allowances, and in the second step, we compared athletes that received partial or total cuts and those who did not. We verified that there were significant differences between athletes with different remuneration statuses (Table 2). In a pair-to-pair comparison, it was verified that athletes who were not paid for their sports activity showed significantly lower levels of stress (22.30 ± 6.13) than the values presented either by athletes who received remuneration (24.02 ± 6.26) or by those who received allowances (25.13 ± 4.68). Among these two last groups, there were no significant differences (*p* > 0.05). Additionally, we found a significant difference between athletes who received cuts in remuneration and those who did not or whose allowances wee reduced (Table 3). For athletes who did not receive cuts in their remuneration (19.88 ± 5.93), the stress perception values were lower than they were for athletes who faced cuts to their remuneration or allowances, either entirely (26.14 ± 4.73) or partially (22.73 ± 5.37). The athletes who did not receive remuneration cuts showed a lower perception of stress values that was not pathological, contrary to the other two groups.

Concerning sleep disorders (Table 2), we verified that athletes who received allowances from their sports activities (3.70 ± 0.62) present with higher sleep disorder values than athletes who received remunerations (3.51 ± 0.68) and athletes that received nothing (3.44 ± 0.85). Additionally, contrary to what was seen for stress, variable wage cuts did not lead to significant differences in the sleep disorder factor (Table 3). Finally, the results show that athletes’ subjective happiness does not depend on whether or not they are paid or receive financial aid (Table 2). Nevertheless, the subjective happiness level (Table 3) varies according to pay cuts. In a pair-to-pair comparison, we verified that athletes that suffered total pay cuts had lower levels of subjective happiness (4.29 ± 1.03) than athletes that suffered only partial cuts (4.76 ± 0.84) or no cuts (5.10 ± 0.87). Although subjective happiness is not affected by whether athletes are remunerated, cuts to wages or allowances impact subjective happiness.

These results permit us to say that H5 (monetary cuts are associated with higher levels of stress and sleep disorders and lower levels of subjective happiness) is partially corroborated.

### 3.2. Mediation Hypothesis Test

We advance to the study of the dynamics between the studied variables, and test H6 (stress levels negatively influence subjective happiness levels) and H7 (sleep quality mediates the relationship between stress and subjective happiness).

The results indicate that stress negatively affects athletes’ subjective happiness (β = −0.497; t(1490) = −15.79; *p* < −0.001). That is, the higher the stress levels of the athletes, the lower their subjective happiness. The R^2^ value of 0.247 was obtained, indicating that stress levels explain 24.7% of the variability in subjective happiness. This model is statistically significant (Table 4). This result shows that H6 (stress levels negatively influence subjective happiness levels) was corroborated.

**Figure 1:**
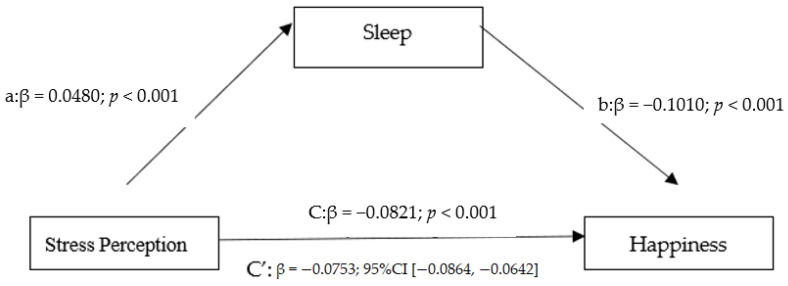
The mediation modeling. Source: authors’ own elaboration based on survey output.

Then, through Figure 1, we can verify that, as required, variable X (stress perception) influences variable M (sleep disorders), and variable M (sleep disorders) influences variable Y (subjective happiness). As previously verified through linear regression, the influence of stress on subjective happiness is also verified (standardized coefficient = −0.4962). Conversely, the indirect effect is significant (a*b: β = −0.0411; 95%CI [−0.0697, −0.0141]). Additionally, the direct effects of perceived stress on subjective happiness with sleep disorders as a mediator variable are also significant, but less significant than the total effect of stress on subjective happiness without sleep disorders as a mediator variable. That is, sleep disorders partially mediate the influences of stress on subjective happiness, which corroborates H7 (sleep quality mediates the relationship between stress and subjective happiness).

The R2 of this mediation model was 0.2564, which indicates that stress levels explain 25.6% of the variability in subjective happiness when mediated by sleep. These results mean that the mediation model is more explicate than the direct model. Additionally, we found that only 13.3% of athletes reported receiving psychological support from their sports clubs. Moreover, 58.8% of athletes say they had no contact with sports clubs during the interruption.

## 4. Discussion

Given these results, we verified that those athletes who saw their training routines and competitions canceled and interrupted manifested with high levels of stress and sleep disorders. These athletes self-report marked breaks in their subjective happiness and were unhappier than before mandatory lockdown. We can suppose that the COVID-19 lockdown and the abrupt interruption to sport activities harmed the psychological health of athletes, and this has profound implications.

Perceived high levels of stress consistently predispose people to illness, which is detrimental to health and performance [40]. Stress is positively related to chronic illness and negatively associated with the functioning of the immune system. The high levels of stress presented by athletes in this study reveal a risk factor, because this stress could cause organic damage and lead to psychological and physical disorders [41]. The consistency between findings using different methodological approaches strongly support the hypothesis that stress is related to headaches, cardiovascular disease, depression, anxiety, and other psychopathologies [42,43]. Sleep disorders also promote these psychopathologies. In our sample, sleep disorders are correlated with stress, which means that stressed athletes do not rest properly and do not have a proper recovery sleep.

Sleep is a biological process that plays a vital role in the normal functioning of the human metabolism. Sleep disorders include deficits in the quantity and quality of sleep and can result in stress responsivity, somatic pain, reduced quality of life, emotional distress, mood disorders, and cognitive, memory and performance deficits. To use a pragmatic definition, “Sleep is an essential component of health and well-being, with significant impacts on physical development, emotional regulation, cognitive performance, and quality of life” [44]. This explains the mediating role that sleep disorders play in stress perception and the subjective happiness relationship.

Additionally, during adolescence, sleep disorders can impact psychosocial health, school performance, and risk-taking behaviors [45,46,47]. In the long term, the consequences of sleep disorders include the greater prevalence of hypertension cases, dyslipidemia, cardiovascular disease, weight-related issues, metabolic syndrome, type 2 diabetes, and colorectal cancer. There is no doubt that sleep disorders have short- and long-term negative consequences for the quality of life and individual health [48].

The risks to the quality of life and health increase over time as these pathological levels persist. In addition, it is not expected that high stress levels will immediately reduce with the start of training and competition. As with sleep disorders, it is unlikely that athletes will immediately return to their standard sleep patterns. This means that pathological stress levels and sleep disorders can affect athletes’ performance and increase the risk of sports injuries on return to competition. 

These results and the possible negative consequences alert us to the need to adequately support athletes in their return to competition. In addition, it is also necessary to help young athletes who are still not allowed to return to competition and are undergoing limited training.

Interruptions to sports competitions and training can be characterized as adverse or negative life events, and we know that positive emotions play an essential role in the recovery process [49]. Given this, the results regarding subjective happiness deserve are also a concern. Subjective happiness is associated with resilience [50]. Resilience is, by definition, “the ability to recover from negative experiences. This mechanism allows for a person to cope with and recover from adverse and stressful situations [51]. As athletes experience breaks in their subjective happiness, they face more difficulties in coping with and recovering from such situations.

The breaks in subjective happiness levels can represent resilience deficits and face more significant difficulties in recovery, giving rise to a break in well-being, life satisfaction and psychological health, as well as limited abilities to develop resources to cope with adverse situations [52]. If perception of stress and sleep disorders have influenced subjective happiness, to lessen this impact, athletes should acquire more adaptive emotional regulation strategies and increase sleep training routines.

Concerning the second aim of this study, it is essential to emphasize that wage cuts can enhance these negative results regarding athletes’ psychological states. It was evident that athletes’ stress and subjective happiness levels were most affected when they suffered wage cuts. Although this situation is negative for all, athletes experiencing cuts and being deprived of sports practice see their livelihood jeopardized. Thus, wage cuts are considered an extra stress factor in this context. This result is consistent with the previous literature, which argues that financial insecurity promotes non-adaptative functioning and is a relevant factor in mental health [18,19].

Moreover, as stated earlier in this paper, this impact can be explained by Maslow’s theory and cognitive theory. Wage cuts can be cognitively assessed as a threat to human needs, regardless of the level of cut (total or partial). As a threat, this will increase stress levels and consequently lower subjective happiness levels.

Furthermore, the scenario presented by this study should alert the responsible entities to the precarious and susceptible situation in which many athletes find themselves, as well as the individual and professional consequences of this situation. The pandemic and the suppression of sports games exposed the precariousness of hourly wage contracts in sporting events [53].

Our study shows that, in Portugal, many athletes are also in precarious and unprotected situations. Additionally, we emphasize that a clear majority of athletes do not have contact with club members or managers, increasing their potential stress factors. We propose that this fact, in addition to pay cuts, can lead to athletes disengaging from their clubs. We challenge future investigations to analyze the relationship between contact with sports clubs during the pandemic and athletes’ commitment to stay or intention to leave.

Finally, regarding the third aim of this study, we conclude that sleep is a mediating factor between stress perception and subjective happiness levels. As expected, stress has a negative influence on subjective happiness and sleep quality. However, we know that these relations are bilateral [22,25]. Improving the quality of sleep will have a positive effect on both stress and subjective happiness.

Recovery sleep is vital for the body to recover from the biopsychological consequences of stress and attenuate the impact of stress on subjective happiness. These results call for the importance of good sleep hygiene training. Good sleep habits can protect athletes mental health of during the pandemic, as well as during competition periods.

The abrupt interruption to competition and sports training negatively affected athletes’ mental health. Athletes saw their daily routines changed. They showed pathological levels of stress and sleep disorders and were less happy than before. This negative state can have an impact on their health and sports performance. Wage cuts made the situation worse, and most sports clubs did not provide psychological support for their athletes. Additionally, during this interruption, sports clubs neglected the contact and relationships with their athletes, and this neglect may have negatively affected future relationships. It is crucial to alert sports clubs to the need to support their athletes during future lockdowns or other crises.

Our research has three main application focuses. First, it is pertinent to impress sports clubs with the importance and possibility of carefully planning their relationship with athletes at moments of withdrawal or forced breaks. Furthermore, to alert the national authorities to the precariousness of sports work and the impact that this situation could have on the mental health of athletes, this situation should be extended to a public health analysis. Conditions for professional athletes should be reviewed. These athletes earn their livelihood, or part of it, in sport. However, their precarious situation allows for them to be left without support in situations such as this, and this fact has a negative impact on their quality of life, livelihood, and mental health.

Second, it is pertinent to relate to coaches, physical trainers, and sports psychologists the relevance of teaching and training athletes for sleep routines as a strategy to improve the biological indicators of stress that can affect performance and the likelihood of injury.

Lastly, we emphasize the need to support athletes during interruptions to training and competition and during their return to competition. Further, we highlight the need to work with athletes on the development of emotional competencies. When generalizing the results, it can be seen society does not have adaptive strategies to cope with significant adverse contexts, and it is necessary to intervene not only to reduce the concerning levels of stress that are currently found, but also to act early and preventatively, providing communities with adaptive coping strategies, emotional competencies, and sleep hygiene training.

## 5. Conclusions

The abrupt cessation of sport activities will have consequences on the level of psychological health of athletes. They face high levels of stress and sleep disorders and a low level of subjective happiness, and self-report a drop in their subjective happiness compared to the moment before lockdown. Additionally, this study concludes that sleep disorders serve as a mediation variable between stress and subjective happiness.

## Figures and Tables

**Table 1:** Means, standard deviations, and Pearson correlation coefficients between variables.

Variable	M	SD	Max.	Min.	1	2	3	4
1. PSS	22.26	6.38	39.00	3.00	1	-	-	-
2. SDS	3.39	0.75	5.00	1.25	0.416 **	1	-	-
3. SHS	4.77	1.04	7.00	1.25	−461 **	−0.290 **	1	-
4. HCI	2.65	1.17	6.00	1.00	−221 **	−0.083 **	0.241 **	1

**Note:** ** *p* < 0.01 PSS—Perceived Stress Scale; SDS—Sleep Disorders Scale; SHS—Subjective Happiness Scale; HCI—Happiness Change Item.

**Table 2:** Comparison levels of stress, sleep disturbance, and subjective happiness among athletes with different pay statuses.

		M	SD	Kruskal—Wallis	*p*
	Remuneration Group	24.02	6.26		
Stress	Allowances Group	25.13	4.68	22.246	0.001
	Non-Remuneration Group	22.30	6.13		
Sleep Disorders	Remuneration Group	3.51	0.68		
	Allowances Group	3.70	0.62	7.001	0.030
	Non-Remuneration Group	3.44	0.85		
	Remuneration Group	4.57	1.05		
Subjective Happiness	Allowances Group	4.56	0.91	1.353	0.508
	Non-Remuneration Group	4.64	1.01		

**Table 3:** Comparison of stress levels, sleep disturbance, and subjective happiness among groups with different levels of pay cuts.

		M	SD	Kruskal—Wallis	*p*
Stress	Total Cut Group	26.14	4.73	32.260	0.001
	Partial Cut Group	22.73	5.37		
	Uncut Group	19.88	5.93		
Sleep Disorders	Total Cut Group	3.61	0.73	2.165	0.339
	Partial Cut Group	3.43	0.88		
	Uncut Group	3.50	0.69		
Happiness	Total Cut Group	4.29	1.03	22.983	0.001
	Partial Cut Group	4.76	0.84		
	Uncut Group	5.10	0.87		

**Table 4:** Linear regression for the effect of stress on athletes’ subjective happiness.

Predictor Variable	Criterion Variable	Z	R^2^	β	t	*p*-Value
Stress Perception	Subjective Happiness	249.433 *	0.247	−0.497 *	−15.793 *	<0.001

**Note:** * *p* < 0.01.

## Data Availability

Not applicable.
